# Therapeutic Implications of Epidermal Growth Factor Receptor (EGFR) in the Treatment of Metastatic Gastric/GEJ Cancer

**DOI:** 10.3389/fonc.2020.01312

**Published:** 2020-08-04

**Authors:** Jacob J. Adashek, Yadis Arroyo-Martinez, Arjun K. Menta, Razelle Kurzrock, Shumei Kato

**Affiliations:** ^1^Department of Internal Medicine, H. Lee Moffitt Cancer Center and Research Institute, University of South Florida, Tampa, FL, United States; ^2^The University of Texas, Austin, TX, United States; ^3^Division of Hematology and Oncology, Center for Personalized Cancer Therapy, Department of Medicine, Moores Cancer Center, University of California, San Diego, La Jolla, CA, United States

**Keywords:** EGFR, next-generation sequencing, targeted therapy, gastric carcinoma, ctDNA

## Abstract

Gastric cancer remains third leading cause of global cancer mortality and is the fifth most common type of cancer in the United States. A select number of gastric cancers harbor alterations in *EGFR* and/or have amplification/overexpression in the HER2; 2–35 and 9–38%, respectively. The advent of next-generation sequencing of tissue and circulating tumor DNA has allowed for the massive expansion of targeted therapeutics to be employed in many settings. There have been a handful of trials using EGFR inhibitors with modest outcomes. Using novel strategies to target multiple co-mutations as well as identifying immunoregulatory molecule expression patterns will potentially drive future trials and improve gastric cancer patient outcomes.

## Introduction

Compared to other cancers, gastric cancer is relatively rare in the U.S. According to the National Institute of Health Surveillance, Epidemiology, and End Results program, stomach cancer comprises 1.6% of all newly diagnosed cancer cases in the U.S (1). However, it is the fifth most common malignancy and the third primary cause of cancer death in the world (2). The highest rates of gastric cancer are in South America, East Asia, and Eastern Europe, and the lowest rates are in Western Europe and the U.S (2). In 2016, there were over 110,000 individuals living with stomach cancer in the U.S. The number of new cases of stomach cancer was 7.4 per 100,000 men and women per year. In 2019, the estimated incidence of gastric cancer will be more than 27,000 with over 11,000 fatalities (1). Although there has been a decrease in the incidence of gastric cancer, the prognosis of patients with advanced gastric cancer continues to be poor, with a median overall survival (OS) of <12 months (3).

To improve the clinical outcome of stomach cancer, molecular sequencing has been done, especially through tissue next-generation sequencing (NGS) and blood-based circulating tumor DNA (ctDNA) (4, 5). The most common alterations that have been seen occur in *TP53* (~51%), *PIK3CA* (~16%), *ERBB2* (~15%), and *KRAS* (~15%) (5). Much of the successes have been seen in targeting HER2 and PD-L1, both of which are FDA-approved (6, 7). However, the efficacy of using a targeted therapy approach for other biomarkers has been limited to date.

One of the potential targets of interest may be epidermal growth factor receptor (EGFR). Although targeting EGFR in gastric cancer has been evaluated extensively and shown to be not efficacious, we have recently demonstrated targeting ctDNA-based *EGFR* amplifications may be a novel target of interest. Herein, we comprehensively review previous experience with anti-EGFR therapies for gastroesophageal cancers and discuss the future direction of personalized therapy.

## Mechanism of EGFR Biology

The molecular mechanism underlying the tumor development, progression, and proliferation in gastric cancer has been mostly associated with tyrosine kinase receptors (RTKs). The most extensively studied RTKs in gastric cancer correspond to the human epidermal growth factor receptor family (ErbB). The most recognized in gastric cancer overexpression are EGFR and HER2. Other recognized tyrosine kinase receptors in gastric cancer include fibroblast growth factor receptor 2 (FGFR2) and MET. In a study by Nagatsuma et al., they reported the percent of overexpression of each tyrosine kinase receptor in patients with gastric adenocarcinoma based on immunohistochemical (IHC) staining. According to their results, various expression patterns were seen; 31.1% for FGFR2, 24.9% for MET, 23.5% for EGFR, and 11.8% for HER2. Of the expression patterns, increased EGFR expression was significantly associated with worse outcomes (8).

The EGFR is a cytoplasmic tyrosine kinase domain composed of a 170,000 kDa transmembrane glycoprotein (9). The ErbB signaling pathway consists of several overlapping and interwoven networks including the phosphatidylinositol 3-kinase (PI3K)/Akt (PKB) pathway, the Ras/Raf/MEK/ERK1/2 pathway, and the phospholipase C (PLCγ) pathway. The PI3K/Akt pathway has an extensive role in cell survival, the Ras/ERK1/2 and PLCγ pathways are both involved in cell proliferation. Along with ErbB signaling, these other pathways influence cell motility, development, cell adhesion, angiogenesis, and organogenesis (2).

Among gastric cancer, 2–35% of cases are reported to have EGFR protein overexpression and/or gene amplification, while 9–38% of cases are reported to have HER2 overexpression (10). Nonetheless, the overexpression of EGFR and HER2 has been demonstrated to significantly impact the prognosis, survival rate, and targeted therapy selection in patients with advances gastric cancer. Using drugs that target specific biomarkers has shown to improve response rates and patient outcomes in multiple lines of therapy (11–14). Also, identifying specific driver mutations in both tissue and ctDNA has allowed for improvements in prognostication as well as treatment strategies (15, 16). Targeted molecular therapy has been the mainstay of treatment in patients with advanced gastric cancer with a goal to increase survival rates and decrease tumor proliferation.

## Trials With EGFR Inhibitors

The correlation between EGFR overexpression and poor prognosis provides a strong rationale for the employment of EGFR targeted therapies combined with standard of care in advanced gastric cancer (17). Various randomized controlled trials (RCT) (mainly without the stratification based on EGFR status) have been conducted to study the efficacy of the adding molecular-targeted therapies to chemotherapeutics for the treatment of advanced gastric cancer (Table 1).

**Table 1:** Trials of EGFR inhibitors in advanced gastric/esophagogastric cancer.

**Combination of EGFR and other pathway inhibitors**						
**Drugs**	**Target**	**Evaluation of EGFR status**	**Status (Phase of trial)**	**Type of cancer**	**Results**	**References**
Matuzumab plus epirubicin, cisplatin, and capecitabine (ECX)	EGFR	Enrolled patients with EGFR positive by IHC	Phase II	Advanced esophagogastric adenocarcinoma	ORR: 31% for ECX/matuzumab vs. 58% for the ECX-alone (*P* = 0.994)	(18)
Panitumumab plus epirubicin, oxaliplatin, and capecitabine (EOC; REAL3 trial)	EGFR	Not tested	Phase III	Advanced esophagogastric adenocarcinoma	Median OS for EOC was 11.3 vs. 8.8 months with mEOC + P (HR = 1.37, 95% CI = 1.07–1.76; *p* = 0.013)	(19)
Cetuximab plus capecitabine and cisplatin (EXPAND trial)	EGFR	Not tested	Phase III	Advanced esophagogastric adenocarcinoma	Median PFS for capecitabine-cisplatin plus cetuximab was 4.4 vs. 5.6 months for capecitabine-cisplatin alone (HR = 1.09, 95% CI = 0.92–1.29; *p* = 0.32)	(20)
Cetuximab (C) plus docetaxel + oxaliplatin (DOCOX)	EGFR	Not tested	Phase II	Advanced esophagogastric adenocarcinoma	Median PFS was 4.7 months for DOCOX (CI = 3.0–5.6) vs. 5.1 months for C + DOCOX (CI = 4.3–5.9)	(21)
					Median OS was 8.5 vs. 9.4 month, 1-year OS rate was 39.1 and 33.0%, ORR was 26 and 38%, respectively, for DOCOX and C + DOCOX	
Panitumumab plus docetaxel, cisplatin, and fluoropyrimidine (ATTAX3 trial)	EGFR	Not tested	Phase II	Advanced esophagogastric adenocarcinoma	RR was 49% in the docetaxel, cisplatin, and fluoropyrimidine arm (CI = 34–64%) and 58% in the Panitumumab plus docetaxel, cisplatin, and fluoropyrimidine arm (CI = 42–72%)	(22)
					Median overall survival was 11.7 months in the chemotherapy arm and 10 months in the combination arm	
Nimotuzumab plus cisplatin and S-1 (NCS)	EGFR	Not tested	Phase II	Advanced esophagogastric adenocarcinoma	ORR for NCS was 54.8 vs. 58.1% for CS alone (*P* = 0.798)	(23)
					Median PFS for CS arm vs. NCS arm (7.2 vs. 4.8 months HR = 2.136; 95% CI = 1.193–3.826; *P* = 0.011)	
					OS for patients in CS arm vs. NCS arm (14.3 vs. 10.2 months; HR = 1.776; 95% CI = 0.972–3.246; *P* = 0.062)	
FOLFOX6 + erlotinib (single-arm study)	EGFR	Not tested for enrollment	Phase II	Advanced esophagus and gastroesophageal junction adenocarcinoma	ORR 45%	(24)
					Median PFS 5.5 months (95% CI = 3.1–7.5 months)	
					Median OS 11 months (95% CI = 8.0–17.4 months)	

*CI, confidence interval; HR, hazard ratio; OS, overall survival; PFS, progression-free survival; PR, partial response; RR, response rate; WT, wild type; ORR, objective response rate*.

In a multicenter, RCT conducted by Rao et al. (18), mastuzumab (anti-EGFR monoclonal antibody) was added to epirubicin, cisplatin, and capecitabine to test the efficacy when treating advanced gastric cancer. In this study, 72 patients with metastatic gastroesophageal cancer with EGFR overexpression by IHC were randomly assigned to either matuzumab plus epirubicin, cisplatin, and capecitabine (ECX) or ECX-alone. Following randomization, 35 patients (median age 59 years old) received ECX and matuzumab while 36 patients (median age 64 years old) received ECX. Adding matuzumab to ECX had no impact on objective response rate (ORR) for ECX/matuzumab compared to ECX-alone (31 vs. 58%, respectively; *P* = 0.994) (18). There was also no significant improvement in median progression-free survival (PFS) for ECX/matuzumab compared to ECX-alone (4.8 vs. 7.1 months, respectively), or in median OS (9.4 vs. 12.2 months, respectively). This randomized, phase II study showed that the addition of weekly matuzumab to ECX made no significant improvement in ORR, PFS, or OS among patients with gastroesophageal cancer with EGFR overexpression.

The REAL3 trial was a study conducted by Waddell et al. (19) in the United Kingdom in which panitumumab (anti-EGFR monoclonal antibody), was added to epirubicin, oxaliplatin, and capecitabine (EOC) in patients with advanced esophagogastric adenocarcinoma (19). In this study, EGFR status was not required for the enrollment. The open-label, multicenter, phase III, RCT enrolled 553 patients to either modified-dose EOC plus panitumumab (EOC + P) or EOC (19). This study reported, a median OS of 11.3 months compared to 8.8 months in 275 patients who received EOC compared to the 278 patients who received EOC + P, respectively (HR = 1.37, 95% CI = 1.07–1.76; *p* = 0.013). The addition of panitumumab was associated with higher rates of grade 3–4 diarrhea, mucositis, rash, and hypomagnesemia (19). Also, the study had four deaths thought to be related to mEOC + P toxicities: septicemia, neutropenic sepsis, pulmonary embolism, and upper gastrointestinal hemorrhage. These findings show that the addition of panitumumab to EOC has no role in unselected advanced esophagogastric adenocarcinoma patients.

The EXPAND trial, another open-label, multicenter, phase III RCT evaluated the addition of cetuximab to capecitabine-cisplatin chemotherapy in patients with unselected advanced gastric or gastro-esophageal junction cancer (20). The study enrolled 904 patients who received capecitabine-cisplatin-alone or in combination with cetuximab (20). For 455 patients who received capecitabine-cisplatin plus cetuximab, the median PFS was 4.4 months compared to 5.6 months for the 449 patients who received capecitabine-cisplatin-alone (HR = 1.09; 95% CI = 0.92–1.29; *p* = 0.32) (20). Grade 3–4 adverse events were reported in this study with 83% in the capecitabine-cisplatin-alone plus cetuximab arm vs. 77% in the capecitabine-cisplatin-alone arm. These events included grade 3–4 diarrhea, hypokalemia, hypomagnesemia, skin reactions, acne-like rash, and hand-foot syndrome. Seventy-two (16%) of four hundred and forty-six patients experienced adverse events that lead to discontinuation of treatment in the cetuximab-containing arm and 80 (18%) of 436 patients in the control group (20). The results of this study suggest that there is no additional benefit of cetuximab to capecitabine-cisplatin compared to capecitabine-cisplatin-alone in the unselected patients in the first-line treatment of advanced gastric cancer.

In another phase II RCT, the addition of cetuximab to docetaxel plus oxaliplatin (DOCOX) was evaluated among metastatic gastroesophageal cancer (21). EGFR status was not part of the inclusion criteria. One-hundred and fifty patients were enrolled and divided into two treatment arms: docetaxel + oxaliplatin (DOCOX) compared to docetaxel + oxaliplatin + cetuximab (DOCOX + C). The patients receiving DOCOX had a median PFS of 4.7 months compared to 5.1 months for those receiving DOCOX + C. The 1-year survival rate for patients randomized to DOCOX was 39.1 vs. 33.0% for those receiving DOCOX + C. With a median OS of 8.5 months for the DOCOX arm vs. 9.4 months for the DOCOX + C arm. The median duration of response for the DOCOX arm was 7.3 months compared to 5.6 months for the DOCOX + C arm (21). Treatment-related adverse events that were grade 3–4 included febrile neutropenia, diarrhea, fatigue, rash, and leukopenia. Based on the study results, the addition of cetuximab to DOCOX did not produce clinically significant outcomes among unselected gastroesophageal cancer. There were no significant improvements in 1-year survival rates, PFS, or OS.

The ATTAX3 phase II trial tested the addition of panitumumab to docetaxel, cisplatin, and fluoropyrimidine (DCF) in EGFR-unselected advanced gastric cancer patients (22). The study enrolled 77 patients from 15 institutions in Australia; 39 patients were randomized to DCF-alone and 38 patients received DCF plus panitumumab. After a median follow-up of 24 months, the median PFS for patients receiving DCF-alone was 6.9 months vs. 6.0 months in the combination arm. For patients receiving DCF-alone, the median OS was 11.7 months compared to 10 months in the combination arm (22). The most common grade 3 or higher adverse events included infection, vomiting, diarrhea, anorexia, and fatigue. This trial revealed similar results when compared to previous clinical trials. There was no meaningful improvement in PFS or OS leading to poor clinically significant outcomes when adding panitumumab to combination chemotherapy regimen among patients with unselected gastroesophageal cancer.

In another open-label, phase II RCT, Du et al. (23) compared the efficacy and safety of nimotuzumab (anti-EGFR monoclonal antibody) plus cisplatin and S-1 (NCS) vs. cisplatin and S-1 (CS)-alone in patients with previously untreated, unresectable, or metastatic gastric cancer. The treatment consisted of 3-week cycles of S-1 and cisplatin with or without weekly nimotuzumab. Sixty two patients were randomized to either NCS or CS-alone. In the 31 patients receiving NCS, the ORR 54.8%, whereas the 31 patient CS-arm, ORR was 58.1% (*P* = 0.798) (23). The median PFS for the CS-arm was 7.2 months compared to 4.8 for the NCS arm (HR = 2.136; *P* = 0.011). Patients in the CS-arm had an OS of 14.3 months vs. 10.2 months in the NCS-arm (HR = 1.776; *P* = 0.062) (23). The authors suggest that there may be a negative interaction between nimotuzumab and S-1, which contributed to the lack of survival benefit for NCS compared to CS-alone. Fewer than 10% of patients in both arms developed grade 3–4 toxicities. The most common grade 3 or higher toxicities were neutropenia, nausea, anorexia, anemia, and thrombocytopenia. The combination of nimotuzumab to CS provided no significant benefit compared to CS-alone in the frontline treatment of unresectable or metastatic gastric cancer.

Lastly, the clinical utility of erlotinib, EGFR tyrosine kinase inhibitor, was evaluated in combination with mFOLFOX6 in patients with metastatic or advanced esophageal and gastroesophageal cancers. This phase II, open label, multicenter study enrolled 33 patients received modified-FOLFOX6 (folinic acid, 5-fluorouracil, oxaliplatin) and erlotinib (24). These patients had an ORR of 51.5% (95% CI = 34.5–68.6%), a median PFS of 5.5 months (95% CI = 3.1–7.5 months), and median OS of 11.0 months (95% CI = 8.0–17.4 months) (24). In all, 16% of the adverse events were grade 3–4 toxicities. The most common grade 3–4 toxicities were: diarrhea (24%), nausea/vomiting (11%), skin rash (8%), and peripheral neuropathy (8%). Although this was a single-arm, non-randomized study it demonstrated that mFOLFOX6 and erlotinib have an acceptable toxicity profile and further studies comparing the combination of erlotinib with mFOLFOX should be considered for further development.

Overall, multiple studies have been conducted with the use of anti-EGFR therapies for gastric cancer patients. However, clinical outcomes have been disappointing. To improve upon these poor outcomes, it may be beneficial to require that patient enrollment be contingent upon having a biomarker (in this case enrolling patients with *EGFR* alterations/overexpression to anti-EGFR regimens). Overall, for the REAL3, EXPAND, DOCOX, ATTAX3, NCS, and FOLFOX6 + erlotinib evaluating the status of patients' EGFR status was not a requisite for inclusion in these studies (19–24). In contrast, the ECX plus matuzumab study included those patients with EGFR positive by IHC (18). Many of the ongoing EGFR inhibitor trials require gastric cancer patients to be tested for *EGFR*-positivity prior to entry into the study (Table 2).

**Table 2:** Ongoing trials of EGFR inhibitors in combination for advanced gastric/esophagogastric cancer.

**Drug**	**Target**	**Evaluation of EGFR status**	**Status (Phase of trial)**	**Type of cancer**	**Results**
**ONGOING CLINICAL TRIALS WITH INHIBITORS THAT BLOCKS EGFR MEMBRANE ASSOCIATION**					
FOLFOX + FOLFIRI + FOLTAX + ABT-806 (anti-EGFR monocolonal antibody) (as part of PANGEA – IMBBP trial)	EGFR	*EGFR* amplification by NGS status required for the enrollment	NCT02213289	Esophagogastric adenocarcinoma	In progress
Intravenous GC-1118 (EGFR inhibitor) in combination with weekly paclitaxel	EGFR	*EGFR* amplification or strong (3+) EGFR immunostaining	NCT04077255	Esophagogastric adenocarcinoma	Not yet recruiting
FATE-NK100 in combination with cetuximab (EGFR inhibitor)	HER2, EGFR	EGFR+ and/or HER2+	NCT03319459	Esophagogastric adenocarcinoma	In process—recruiting
Varlititib (EGFR inhibitor) + mFOLFOX6	EGFR, HER2, HER4	IHC and FISH	NCT03130790	Gastric	Phase II/III

*CR, complete response; CI, confidence interval; HR, hazard ratio; NGS, next-generation sequencing; OS, overall survival; PFS, progression-free survival; PR, partial response; RR, response rate; WT, wild type; ORR, objective response rate*.

Ongoing clinical trials of EGFR inhibitors continue to play a critical role in the evaluation of efficacy, safety profile, and overall response and survival rates in patients with advanced gastric cancer. The following ongoing trials focus on the evaluation of combination chemotherapy with targeted anti-EGFR antibodies: FOLFOX + FOLFIRI + FOLTAX + ABT-806 (NCT02213289), intravenous GC-1118 in combination with weekly paclitaxel (NCT04077255), FATE-NK100 in combination with cetuximab (NCT03319459), and varlititib + mFOLFOX6 (NCT03130790). All trials are in the process of recruitment (Table 2). All of these ongoing trials use a genomic (NGS, FISH) or proteomic biomarker (IHC, immunostaining), it is unclear which of these modalities will fare as the most optimal biomarker for EGFR-positive gastric cancer; however, using some form of selection stratification is imperative in best identifying an inclusion biomarker in these patients.

## Future Directions

**Figure 1:**
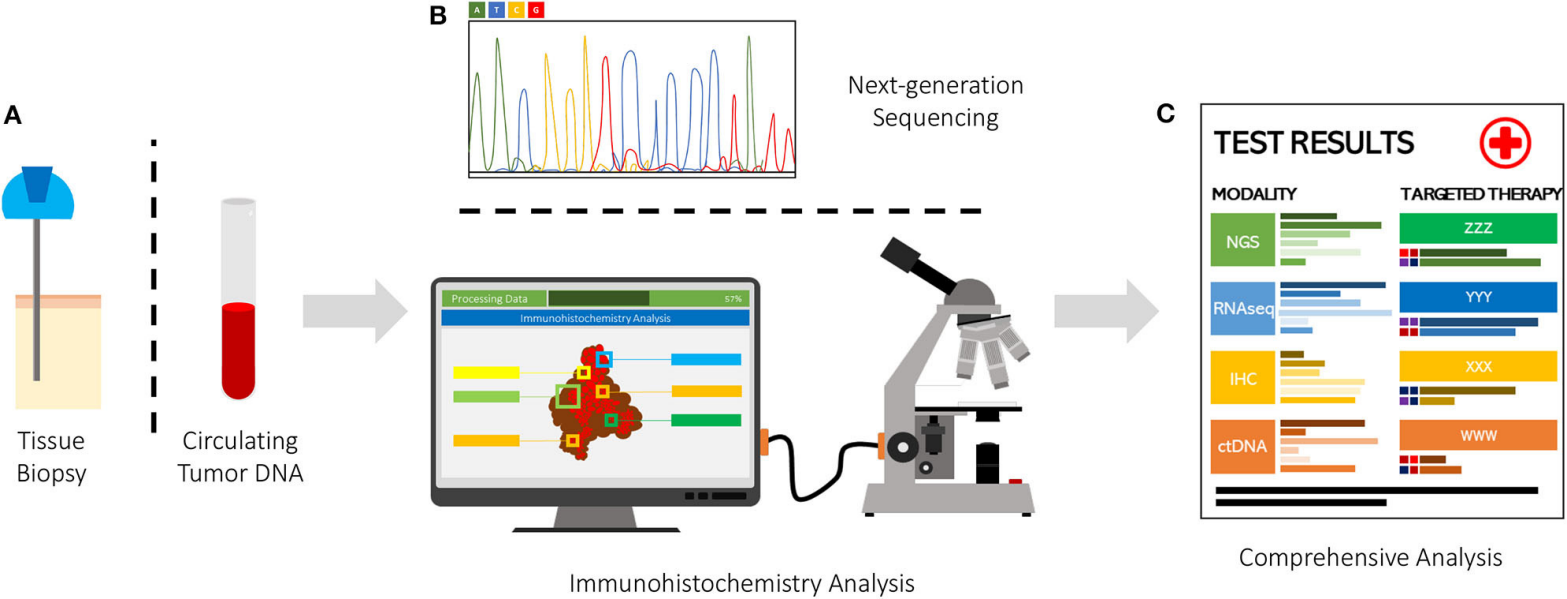
Various modality tumor genome-transcriptome-proteome analysis. **(A)** Tissue sampling via biopsy or peripheral blood sample. **(B)** Modality for tissue analysis via DNA, RNA, protein. **(C)** Results of identified biomarker in NGS, RNAseq, IHC, ctDNA for targeted therapy approach.

There are now at least six completed trials using an EGFR inhibitor alone or in combination with chemotherapeutics for patients with advanced esophagogastric cancers. The use of combination chemotherapy with targeted therapies may continue to be less fruitful than hoped. Important to note that most studies were conducted where patients were not selected based on EGFR status. This outcome although disappointing, is not surprising with meta-analysis showing that giving various types of targeted therapies among unselected patients, the response rate is ~5%, but if select for a genomic target, the response rates can be up to ~42% (11–13). We recently evaluated *EGFR* amplification status by ctDNA from over 28,000 patients with diverse malignancies using clinical-grade NGS (25). In this study, ~8% of patients harbored an *EGFR* amplification in their ctDNA, with *EGFR* amplifications being most common in colorectal cancer (16% of patients), NSCLC (9%), genitourinary cancers (8%), cutaneous tumors (7%), and breast malignancies (7%) (6). Anti-EGFR–based therapies among patients found to have *EGFR* amplification by ctDNA analysis achieved responses in ~55% of patients (including patients with gastric cancer). Therefore, further investigation is warranted on the use of EGFR inhibitors in patients with *EGFR* amplification in ctDNA. Using technologies such as NGS and its application to ctDNA will likely guide treatments and offer a multigene-targeted approach in advanced gastric cancer. Additionally, identifying co-expressed immunoregulatory molecules may also offer potential novel strategies for certain patients and add to the treatment artillery (26–28). Use of artificial intelligence technology to rapidly and objectively analyze immunohistochemistry staining of immunoregulatory patterns may also inform potentially beneficial combinations of gene- and immune- targeted therapeutics (29). There is still also considerable room for improvement in treating EGFR-positive gastric cancers; however, the recognition of the role of NGS, machine learning/artificial intelligence, and combination strategies will hopefully continue to improve survival (Figure 1).

## Author Contributions

JA, YA-M and SK contributed to writing portion of manuscript. AM created figure. All authors approved final draft of manuscript. All authors contributed to the article and approved the submitted version.

## Conflict of Interest

RK has stock and other equity interests in IDbyDNA, CureMatch, Inc., and Soluventis; has a consulting or advisory role for Gaido, LOXO, X-Biotech, Actuate Therapeutics, Roche, NeoMed, and Soluventis; has received a speaker's fee from Roche; has received institutional research funding from Incyte, Genentech, Merck Serono, Pfizer, Sequenom, Foundation Medicine, Guardant Health, Grifols, Konica Minolta, and OmniSeq; is a Board Member for CureMatch, Inc and CureMetrix Inc; and has received research funding from the Joan and Irwin Jacobs Fund, and the National Cancer Institute, grant number P30 CA023100. The remaining authors declare that the research was conducted in the absence of any commercial or financial relationships that could be construed as a potential conflict of interest.
